# The role of age and physical fitness on the relationship between physical activity and executive function

**DOI:** 10.1017/S1355617725101446

**Published:** 2025-08

**Authors:** Matthew Stauder, Olivia Horn, Scott M. Hayes

**Affiliations:** 1 Department of Psychology, The Ohio State University, Columbus, OH, USA; 2 Chronic Brain Injury Program, The Ohio State University, Columbus, OH, USA

**Keywords:** aging, cardiorespiratory fitness, cognition, lifestyle, speed, strength

## Abstract

**Objective:**

Few studies examine the relationship between physical activity, multiple physical fitness domains (cardiorespiratory fitness, strength, speed), and cognition. Our objective was to investigate the association between physical activity and executive function in middle-aged and older adults and examine whether modifiable physical fitness components explain the relationship between physical activity and cognition.

**Method:**

Self-reported moderate-to-vigorous physical activity and objective measures of cardiorespiratory fitness (2-minute walk test), strength (grip strength), speed (4-meter walk test), and executive function were collected from 623 adults within the Human Connectome Project–Aging (ages 36 – 100 years; mean = 59.2 years; 57.8% female). Relative importance metrics, multiple regression, and conditional process analysis were used to examine relationships of age, physical activity, and physical fitness with executive function.

**Results:**

Greater physical fitness was related to better executive function performance (*β* = 0.28, *p* < .001). Physical activity was not associated with executive function (*β* = −0.04, *p* = .16). There was an indirect relationship between physical activity and executive function through physical fitness (ab = 0.02, 95% CI: 0.004 – 0.04). This association was explained primarily by the indirect association of cardiorespiratory fitness with physical activity and executive function. The indirect association of cardiorespiratory fitness with physical activity and executive function was significant in older study participants (mean (59 years) and + 1 SD (74 years)), but not younger (−1 SD (44 years)), although between-group comparisons were not significant.

**Conclusions:**

These data highlight potential differential associations with cognition when considering physical activity and physical fitness, and the importance of considering multiple domains of physical fitness in relation to physical activity and cognitive performance.

## Statement of Research Significance

**Research Question(s) or Topic(s):** Executive function refers to a constellation of abilities, including cognitive flexibility, planning, manipulating information in one’s mind, and inhibiting distracting information. Executive function declines with age. Here we examined how physical activity and different types of physical fitness were related to executive function and aging. **Main Findings:** Physical fitness, but not physical activity, was linked to executive function, regardless of one’s age. Physical activity was linked to cardiorespiratory fitness and grip strength, which in turn were associated with better executive function. The indirect link between physical activity, cardiorespiratory fitness, and executive function was significant in the older participants, but not the youngest participants, although the strength of the effect was not significantly different between the age groups. **Study Contributions:** Our study demonstrates that different fitness domains have different associations with executive function. Maintaining cardiorespiratory fitness and strength with physical activity is likely important to maintain executive function abilities.

## Introduction

One domain of cognition susceptible to decline with age is executive function, which refers to effortful, higher-level processes involved in goal setting, information updating and monitoring, inhibition, and the top-down control of other cognitive abilities (Diamond, 2013; Miyake et al., 2000). Executive function serves a vital role in motivation, emotion regulation, inhibiting behavioral impulses or prepotent responses, and psychosocial functioning (Luszcz, 2011; Reuter-Lorenz et al., 2021). Executive function has been associated with health-related quality of life (Laera et al., 2023) and instrumental activities of daily living among older adults (Brothers & Suchy, 2022), highlighting the need to identify the extent to which modifiable lifestyle factors are associated with executive function in older adulthood.

One modifiable lifestyle factor associated with better cognitive function among older adults is physical activity. Physical activity describes any bodily movement produced by skeletal muscles that results in energy expenditure above basal levels (Vanhees et al., 2005). Across adult age groups, higher physical activity levels have been associated with better cognitive performance cross-sectionally (Cox et al., 2016; Erickson et al., 2019). Longitudinal observational research has shown that middle-aged and older adults who self-report engaging in greater amounts of physical activity are at a reduced risk of future cognitive decline and dementia (Blondell et al., 2014). The most robust support for an association between moderate-to-vigorous intensities of physical activity and improvements in tasks of executive functions is typically observed in samples of older adults (Erickson et al., 2019; O’Brien et al., 2021). However, there is considerable heterogeneity in the magnitude of the association (Erickson et al., 2019), and limited research has explored these relationships among middle-aged adults, but see Cox et al. (2016) and Wang et al. (2022). Moreover, additional research is needed to clarify the mechanisms contributing to the heterogeneity of the association of physical activity with cognitive performance across middle-aged and older adults.

One such mechanism is physical fitness, which refers to physical attributes that are influenced by engagement in physical activity and are associated with general health and reduced risk of chronic disease (Caspersen et al., 1985; Vanhees et al., 2005). Aspects of physical fitness, such as cardiorespiratory fitness, have been positively associated with executive function performance among older adults (S. M. Hayes et al., 2016; Kawagoe et al., 2017; Predovan et al., 2021), even when controlling for the effect of concurrent physical activity (Nilsson et al., 2022). The benefits of cardiorespiratory fitness on fluid cognition have also been found in young adults (Won et al., 2024), but samples including middle-aged adults are scarce. In a middle-aged to older-adult sample, Callow & Smith (2023) reported that cardiorespiratory fitness, but not self-reported total physical activity level, was associated with tasks of processing speed and fluid intelligence. However, physical fitness is a multidimensional construct, and other domains of physical fitness beyond cardiorespiratory fitness, such as muscular strength, have also been associated with cognitive performance. For instance, stronger grip strength has been associated with better performance in memory, processing speed/attention, and reasoning tasks (Sprague et al., 2019). Speed is a skill-related component of fitness and refers to one’s ability to perform a movement within a short period of time (Caspersen et al., 1985; Vanhees et al., 2005). Here, gait speed was considered a proxy for speed. Similar to health-related fitness components, such as cardiorespiratory fitness and strength, gait speed can be improved with physical activity (Van Abbema et al., 2015). Slow gait speed and declines in gait speed over time have been associated longitudinally with health-related outcomes, such as cognitive decline (Mielke et al., 2013) and risk of dementia (Dumurgier et al., 2017). Studies have found associations between gait speed and cognition amongst older adults (Stauder et al., 2024), including cross-sectional and longitudinal relationships with a diverse set of executive function tasks (Kearney et al., 2013; Wu et al., 2023).

Physical activity improves physical fitness (Ferreira et al., 2012; Liberman et al., 2017), and multiple components of physical fitness have been associated with executive function performance (S. M. Hayes et al., 2016; Sprague et al., 2019; Wu et al., 2023). Thus, physical fitness may be an important link in physical activity-cognition relationships. However, gaps in the literature remain regarding the relative contributions of multiple simultaneously assessed domains of physical fitness on cognition and whether these relationships differ across the lifespan, despite calls from authors to investigate the association between physical activity and cognitive ability in understudied groups such as middle-aged adults (Prakash et al., 2015). Few studies have examined physical activity and executive function relationships across middle age and older adulthood – but see Callow & Smith (2023) and Eppinger-Ruiz de Zarate et al. (2024) – and studied the role of physical fitness components as mediators in the relationship between engagement in physical activity and executive function performance – but see Castells Sánchez et al. (2021). Moreover, although processing speed has been proposed as a single mechanism that may account for age-related performance differences across multiple cognitive domains (Albinet et al., 2012; Salthouse, 1996), few studies have controlled for processing speed when examining the relationship between fitness or physical activity and executive function. This approach allows for greater confidence that observed associations are related to the specific cognitive construct of interest (i.e., executive function) rather than processing speed.

The purpose of this study was to examine age as a boundary condition and physical fitness as a plausible link in the relationship between physical activity and executive function performance in a sample of community-dwelling, middle-aged to older adults from the Lifespan Human Connectome Project – Aging (HCP-A). This study had three aims: 1) to examine the relative importance and magnitude of the direct associations of physical activity and physical fitness on executive function performance compared to other variables known to be associated with executive function such as age and processing speed, 2) to investigate the interaction of physical activity and physical fitness with age on executive function performance, and 3) to explore the relative indirect relationship of physical activity on executive function performance via their associations with physical fitness metrics such as cardiorespiratory fitness, strength, and speed.

## Method

### Participants

Data for this project were obtained from the HCP-A Lifespan 2.0 Release database (https://www.humanconnectome.org/study/hcp-lifespan-aging/data-releases). HCP-A was launched in 2017 with the goal of developing a publicly available database of brain, cognitive, and biometric data in a large, representative sample of healthy American adults. The latest HCP-A data release contained cross-sectional data from 725 participants recruited from four acquisition sites across the United States. Study participants were adults who provided informed consent and were aged 36 years or older at time of enrollment. Descriptions of the recruitment strategy and full exclusion criteria for study participants are available elsewhere (Bookheimer et al., 2019).

Of the 725 participants in the dataset, 633 had complete physical activity and cognitive data. Eight participants were excluded due to low accuracy scores on the Flanker or Dimensional Change Card Sort cognitive tasks; that is, accuracy was not ≥ 80% correct, which is required to generate the summary scores for Flanker and Dimensional Change Card Sort. One outlier was removed for low composite cognitive performance and one outlier was removed for high cardiometabolic risk (composite scores ≥ 3 standard deviations from sample mean). Therefore, the final sample included 623 adults aged 36 to 100 years. See Table 1 for participant characteristics.

**Table 1.** Sample demographic and clinical characteristics (*n* = 623).

Measure (unit)	Mean (SD)	Min – Max
Age (years)	59.2 (15.0)	36 – 100
Gender (*n* and % female)	360 (57.8)	
Education (years)	17.5 (2.2)	7 – 21
Race (*n* and %)		
White	445 (71.4)	
Asian	48 (7.7)	
Black	88 (14.1)	
American indian	2 (0.3)	
Multiracial	27 (4.3)	
Not reported	13 (2.1)	

Cognitive status (Montreal Cognitive Assessment ≥ 23) (*n* and %)		
Cognitively normal	567 (91.0)	
Mild cognitive impairment	56 (9.0)	

Weekly moderate-to-vigorous physical activity (metabolic equivalents of task)	1799 (2204)	0 – 12480

Systolic blood pressure (mm Hg)	129.3 (16.3)	79 – 189
Diastolic blood pressure (mm Hg)	79.5 (10.4)	51 – 114
Serum glucose (mg/dL)	101.7 (15.5)	64 – 249

2-Minute walk test (ft)	607.4 (101.6)	194 – 907
Grip strength (lbs)	74.6 (24.7)	21.4 – 168.1
Gait speed (m/s)	1.3 (0.3)	0.5 – 2.4

Processing Speed Comparison Test total score	43.1 (9.0)	16 – 101
Flanker composite score	8.0 (0.8)	4.1 – 10.0
Dimensional Change Card Sort composite score	8.2 (0.9)	5.4 – 10.0
List Sort Working Memory Test total score	16.8 (3.0)	7 – 26

#### Demographics

Demographic information, including age, sex, race, and years of education, was self-reported during the intake interview.

#### Cardiometabolic risk

To control for cardiometabolic contributions to brain health, risk factors for the development of cerebrovascular disease were assessed via vital signs and blood samples. A modified cardiometabolic risk score was developed from available variables known to increase risk for cardiovascular disease, namely systolic and diastolic blood pressure and serum glucose levels (Smith, 2007). Each variable was transformed into a *z*-score [(x – x̄)/sd] and then averaged to generate a composite measure of cardiovascular disease risk, with higher scores indicating greater risk.

#### Physical activity

Physical activity was assessed with the International Physical Activity Questionnaire – Short Form (IPAQ-SF; Craig et al., 2003). Participants self-reported the number of days per week and average daily time spent performing moderate and vigorous intensity activities. Examples of moderate activities provided by the IPAQ included carrying light loads and bicycling at a regular pace. Examples of vigorous activities included aerobics, heavy lifting, or fast bicycling. Self-reported daily activities with a duration of greater than 180 minutes were truncated to 180 minutes according to recommended data processing methods (Sjostrom et al., 2005). Weekly moderate and vigorous intensity physical activity minutes were multiplied by a corresponding Metabolic Equivalent of Tasks (MET) value: Moderate PA = 4.0 METs and Vigorous PA = 8.0 METs. Therefore, total weekly moderate and vigorous physical activity scoring in MET-minutes/week was calculated as follows:

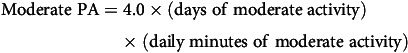




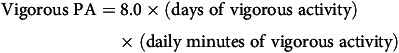




MET-minutes/week in each intensity domain were transformed into *z*-scores [(x – x̄)/sd] and averaged, creating a measure of total weekly moderate-to-vigorous physical activity (MVPA).

#### Physical fitness

##### Cardiorespiratory fitness

Sub-maximal cardiorespiratory fitness was assessed with the NIH Toolbox 2-minute walk test (Reuben et al., 2013). Participants were asked to walk as fast as they could on a flat surface back and forth between two cones set 50 feet apart for two minutes. Greater distance in feet covered in two minutes indicated greater cardiovascular endurance, a proxy for cardiorespiratory fitness.

##### Muscular strength

Muscular strength was assessed with the NIH Toolbox grip strength dynamometry test (Reuben et al., 2013). Participants were seated in a chair with their feet touching the ground. With their elbow bent at 90 degrees, arm near their trunk, and wrist in a neutral position, participants used their hand to squeeze a Jamar Plus Digital dynamometer as hard as possible for three seconds. After a sub-maximal practice trial, one test trial was completed on each hand, and the maximum value between the two trials was chosen.

##### Speed

Preferred gait speed was assessed with the NIH Toolbox 4-meter walk test (Reuben et al., 2013). Participants were asked to walk between two markers set four meters apart at their usual pace. Participants completed one practice trial, followed by two timed trials. Gait speed was calculated by dividing 4 meters by the fastest time to complete the timed walk (meters/second), with higher values indicating faster gait speeds.

#### Cognitive function

Cognitive abilities were assessed with the original version of the NIH Toolbox Cognition Battery (Weintraub et al., 2013). We used raw scores from the cognitive tasks to minimize missing data and to avoid overcorrection for demographic variables in our models.

##### Inhibition

Inhibition was assessed with the Flanker Inhibitory Control and Attention Test (Flanker; Weintraub et al., 2013). Participants viewed a row of stimuli (directional arrows) and responded based on the direction of the central stimulus (arrow) while inhibiting attention to surrounding stimuli (other arrows), which may be congruent (← ← ← ← ←) or incongruent (← ← → ← ←) with the direction of the central arrow. The computed score for the Flanker subtest, which combines accuracy and response time performance, was selected as the outcome measure. Scores range from 4 to 10, with higher scores indicating greater inhibition ability.

##### Working memory

During the List Sorting Working Memory Test (Weintraub et al., 2013), pictures of different foods and animals were sequentially displayed with their name on the iPad, along with an accompanying audio recording that named the stimulus. After each trial, the participant was tasked with verbally repeating the items back in size order from smallest to largest, first within a single dimension (either foods or animals; 1-list) and then on two dimensions (mixed encoding list of foods and animals; 2-list). The total score, summing the total number of items correctly recalled and sequenced across the 1-list and 2-list tasks, was selected as the outcome measure. Scores range from 0 to 26, with higher scores indicating greater working memory ability.

##### Cognitive flexibility

During the Dimensional Change Card Sort Test (Weintraub et al., 2013), participants were presented with pictures that vary along two dimensions (shape and color) and were tasked with matching a series of bivalent test pictures to the target pictures based on the randomly switching dimensions. The computed score, combining response time and accuracy, was selected as the outcome measure. Scores ranged from 4 to 10, with higher scores indicating higher levels of cognitive flexibility.

##### Processing speed

During the Pattern Comparison Processing Speed Test (Weintraub et al., 2013), participants decided whether two pictures presented beside one another were the same or not as quickly as possible. The total number of patterns correctly discerned in the 85 seconds allotted was selected as the outcome measure. Higher scores indicate greater processing speed performance.

##### Cognitive status

Assessed with the Montreal Cognitive Assessment (MoCA; Nasreddine et al., 2005), cognitive status was binarized using a cut-off score of ≥ 23 indicating cognitively normal as recommended by a systematic review and meta-analysis of diagnostic validity studies of MoCA cut scores (Carson et al., 2018) and included as a covariate.

### Data analyses

All analyses were conducted using R Statistical Software (v4.1.1; R Core Team, 2021). Continuous covariates (age, education, cardiometabolic risk, processing speed) were transformed into *z*-scores using the mean and standard deviation from the final sample. Composite scores of physical fitness and executive function performance were calculated for each participant to increase the reliability of our results and generalizability to extant literature. Performance on each of the component tasks was *z*-scored [(x – x̄)/sd] and averaged to generate composite measures of physical fitness and executive function, with higher scores indicating better performance. Therefore, results from all models are presented as standardized beta coefficients, representing the *β*-value standard deviation change in executive function composite for every one standard deviation change in the predictor variable, to aid in the interpretation of effect sizes. We used a confirmatory factor analysis to verify that the three physical fitness components loaded onto a latent construct representing physical fitness. All three fitness variables had significant factor loadings, with the latent fitness variable explaining 81.2% of variance in cardiorespiratory fitness, 26.0% of variance in grip strength, and 17.6% of variance in gait speed. Similarly, we used a confirmatory factor analysis verifying that the three executive function components loaded onto a latent construct representing executive function. All three executive function tasks had significant factor loadings, with the latent executive function variable explaining 63.9% of variance in cognitive flexibility, 67.3% of variance in inhibition, and 19.8% of variance in working memory.

Complete data were necessary for the analyses. Therefore, missing data were imputed with multivariate imputation using predictive mean matching for continuous data with the Multivariate Imputation for Chained Equations (MICE) package in R (Buuren & Groothuis-Oudshoorn, 2011). MICE imputes incomplete data using fully conditional specification, whereby an iterative algorithm generates plausible values for an incomplete column using all other columns in the dataset as sets of predictors. Therefore, MICE attempts to maintain existing relationships in the dataset. Two participants had physiologically implausible values for their diastolic blood pressure (mmHg = 27) and serum glucose (mg/dL = 310); therefore, their values were omitted prior to imputation. Percent of missing data imputed by variable is presented in Supplemental Table 1. Overall, 1.9% of values in the dataset were missing prior to imputation and only one variable (serum glucose) was missing > 5% of data. Imputation did not alter sample means for any variable with imputed data (*p*’s = .49 – .99). Complete cases did not differ from those with imputed data on age, sex, education, cognitive status, or cardiometabolic risk, but completers had higher reported physical activity, cardiorespiratory fitness, and executive function performance and were trending as less racially diverse. Only participants with complete cognitive data were selected; therefore, no outcome measures were imputed.

#### Relative importance and independent relationships of age, physical activity, and physical fitness to executive function performance

##### Relative importance for linear regression (relaimpo)

Relative importance refers to the quantification of an individual regressor’s contribution to a multiple regression model by considering both its direct effect (correlation with outcome) and its effect when combined with other variables in the model (Groemping, 2007). We used analyses of relative importance to examine the relative strength of associations between physical activity, physical fitness, and covariates with executive function performance. Relative importance metrics – such as the Lindeman, Merenda, and Gold (lmg) analysis of partitioned variance – from the *relaimpo* R package (Groemping, 2007) quantify average regressor contributions to a multiple linear regression model outcome, controlling for collinearity and order of entry of variables into the model. The *relaimpo* analysis allowed for data-driven insight into those variables accounting for the most variance in executive function performance and minimizing the number of statistical models or reliance on arbitrary selection of which variables to examine as variables of interest.

##### Hierarchical multiple linear regression

We used multiple regression analyses to test the associations between physical activity and physical fitness with executive function performance controlling for covariates (age, sex, race, years of education, cardiometabolic risk, cognitive status, and processing speed performance). All covariates were included in the final model, as they were significantly correlated with the executive function composite prior to or after adjustment for other covariates. The full multiple regression model from the *relaimpo* analysis was used to explore the magnitude and significance of independent relationships between variables and executive function performance. To determine the independent contributions and amount of unique explained variance, two follow-up models included physical activity and physical fitness in separate final steps of the hierarchical multiple regression.

#### Relationship of physical activity and physical fitness with executive function performance across age

To assess if the associations between physical activity and physical fitness with executive function performance were dependent on age, two bootstrap moderation regression models were analyzed using the PROCESS macro in R (A. F. Hayes, 2023). Each model tested for an interaction between the variable of interest and age on executive function performance, controlling for all covariates. Conditional relationships were estimated using bootstrap confidence intervals from 5000 bootstraps. Bootstrap resampling was implemented as it provides more reliable estimates of standard errors and confidence intervals, it does not rely on assumptions about the population distribution of the interaction effect, and it is more robust to outliers than traditional regression (A. F. Hayes, 2023). Confidence intervals of the interaction term entirely above or below 0 indicated a significant moderating relationship. The interactions between physical activity and physical fitness with age were visualized by plotting the association of variable of interest on executive functions across the 16^th^ (44.4 years), 50^th^ (59.2 years), and 84^th^ (74.1 years) percentiles of the sample’s age. These percentiles are implemented by PROCESS as they represent one standard deviation below the mean, the mean, and one standard deviation above the mean in a normal distribution but are less affected by any skew in the distribution.

#### Indirect relationships between physical activity and executive function performance through physical fitness

To investigate if physical activity related to executive function performance indirectly via composite physical fitness or its components, ordinary least squares regression mediation analyses were conducted using the PROCESS macro available in R (A. F. Hayes, 2023). We used a simple mediation model to test if there was an indirect association between physical activity and executive function via physical fitness and a multiple mediation model to test if there was an indirect association via any of the components of physical fitness, controlling for the other two. This approach does not require a preliminary establishment of a statistically significant total effect of an independent variable of interest on the outcome variable and is aligned with recommendations regarding the use of mediation analysis in psychological research (A. F. Hayes, 2023; Igartua & Hayes, 2021). Indirect relationships were estimated using bootstrap confidence intervals from 5000 bootstraps. Confidence intervals of the indirect relationship entirely above or below 0 indicated a significant positive or negative indirect relationship, respectively. Follow-up conditional process analyses were used to test if any observed indirect relationships were dependent on age. These models examined the indirect relationships between physical activity and executive function via composite physical fitness, or physical fitness components, across the 16^th^ (44.4 years), 50^th^ (59.2 years), and 84^th^ (74.1 years) percentiles of age.

## Results

### Relative contributions and independent relationships of age, physical activity, and physical fitness to executive function performance

#### Relative importance of independent variables (relaimpo)

**Figure 1.**
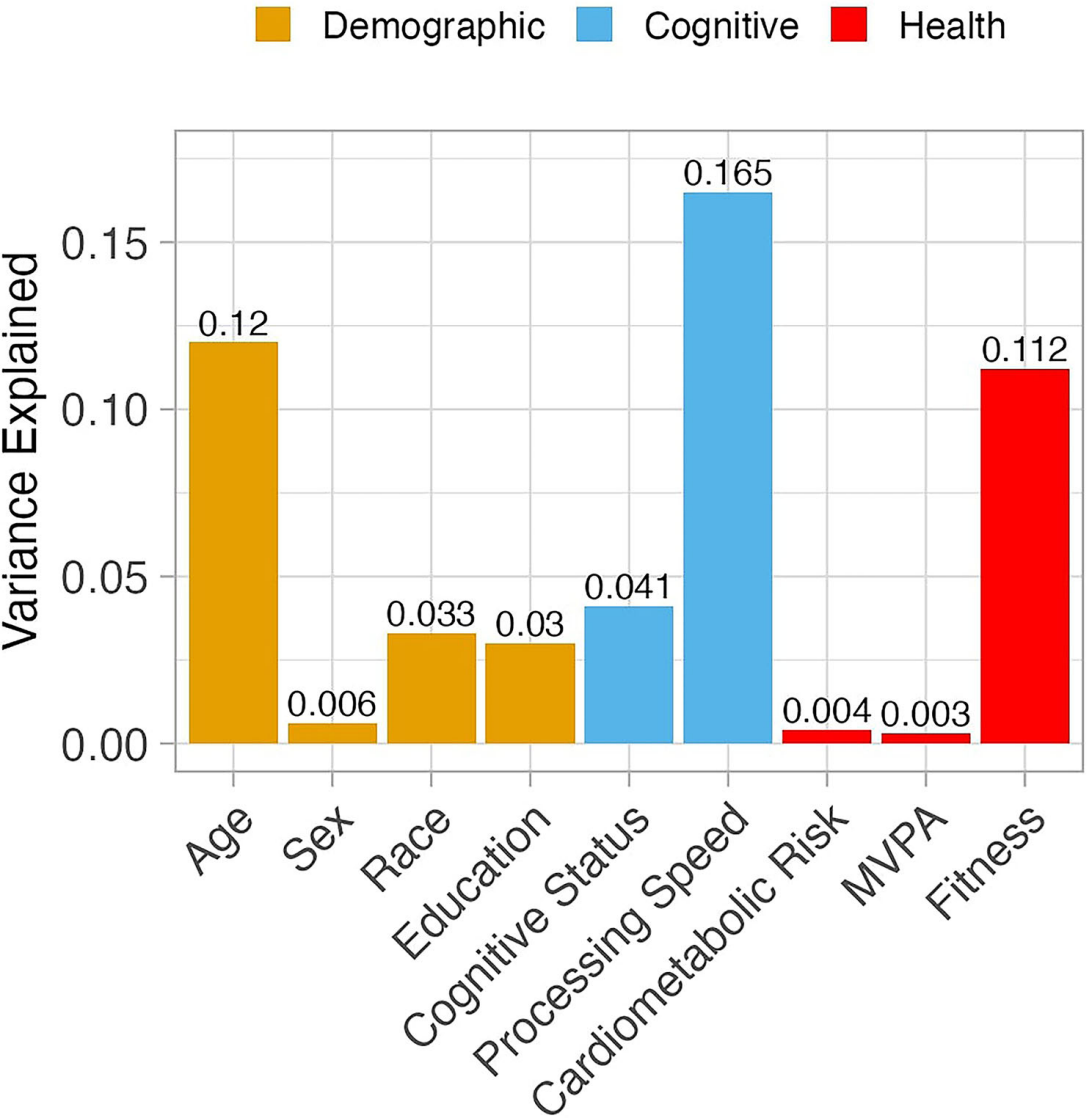
Relative importance of demographic, cognitive, and health variables to executive function performance.

Metrics of relative importance for all variables to executive function performance are presented in Figure 1. Consistent with prior literature, age (.12 proportion of variance) and processing speed performance (.165) had relatively important relationships with executive function performance. Participant self-reported engagement in MVPA (.003) was not a relatively important contributor in the model, whereas composite physical fitness (.112) explained a roughly similar proportion of variance to age and processing speed. Cardiometabolic risk (.004) did not have a relatively important relationship with executive function.

#### Multiple linear regression

Multiple linear regression output from the full model used for *relaimpo* analysis examined the strength and significance of independent variables with executive function performance. The overall model was significant (*F*(13, 609) = 49.47, *p* < .001), accounting for 51.4% of variance. Increased age (*β* = −.20, *p* < .001), identifying as Black or African American (*β* = −.37, *p* < .001), identifying as male (*β* = −.11, *p* = .04), and scoring < 23 on the MoCA (*β* = −.42, *p* < .001) were associated with lower executive function performance. Higher levels of educational attainment (*β* = .10, *p* < .001) and better processing speed performance (*β* = .24, *p* < .001) were associated with better executive function performance. There was not an independent relationship between reported MVPA and executive function (*β* = −.04, *p* = .16). However, higher levels of composite physical fitness were associated with better executive function performance (*β* = .28, *p* < .001). Using hierarchical regression, entering covariates (including physical fitness) in Step 1 (R^2^ = .512, *F*(12, 610) = 53.34, *p* < .001) and adding MVPA in Step 2 (R^2^ = .514, *F*(13, 609) = 49.47, *p* < .001) did not improve the model fit (**Δ**R^2^ = .002, *F*(1, 609) = 2.02, *p* = .16). In contrast, in a separate hierarchical regression including MVPA in Step 1 (R^2^ = .474, *F*(12, 610) = 45.77, *p* < .001), adding physical fitness in Step 2 (R^2^ = .514, *F*(13, 609) = 49.47, *p* < .001) explained significant additional variance in executive function performance (**Δ**R^2^ = .04, *F*(1, 609) = 49.92, *p* < .001). The effects were replicated in an analysis with cases with missing data excluded rather than imputed.

### Relationships of physical activity and physical fitness with executive function performance across age

**Figure 2.**
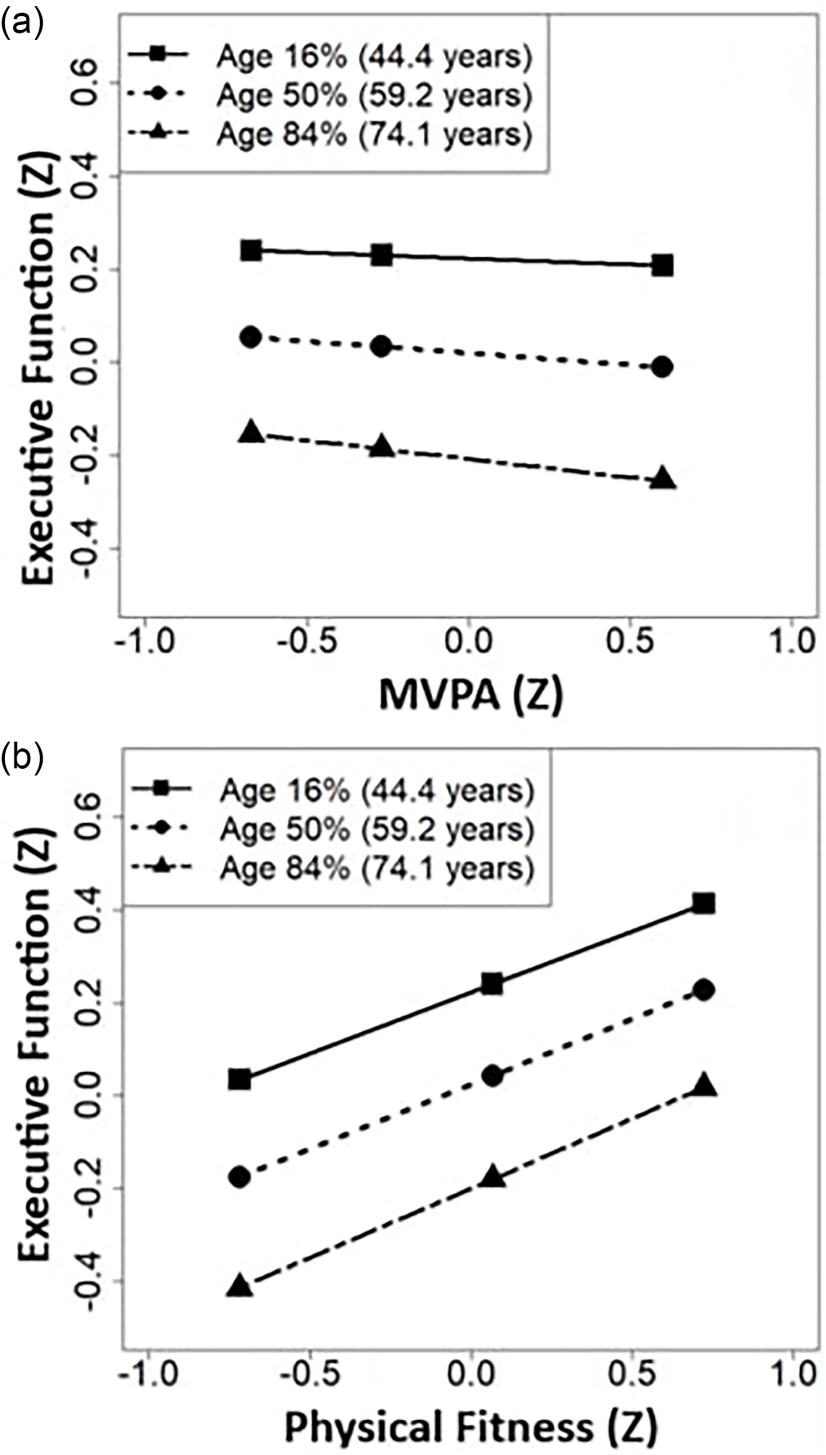
(a) Relationship between moderate-to-vigorous physical activity (MVPA) and executive function at different ages. (b) Relationship between physical fitness and executive function at different ages.

The relationships of physical activity and physical fitness with executive function performance are plotted across age values in Figure 2. Overall, there was not a relationship between self-reported MVPA and executive function, and that relationship was not conditional on participant age (**Δ**R^2^ = .0006, Bootstrap CI: -0.07 – 0.03, *F*(1, 612) = .70, *p* = .40). In contrast, there was a positive relationship between physical fitness and executive function, and the association did not interact with participant age (**Δ**R^2^ = .0003, Bootstrap CI: −0.04 – 0.07 *F*(1, 612) = .32, *p* = .57). The effects were replicated in an analysis with missing data excluded rather than imputed.

### Indirect relationships between physical activity and executive function performance through physical fitness

#### Simple mediation with composite physical fitness

**Figure 3.**
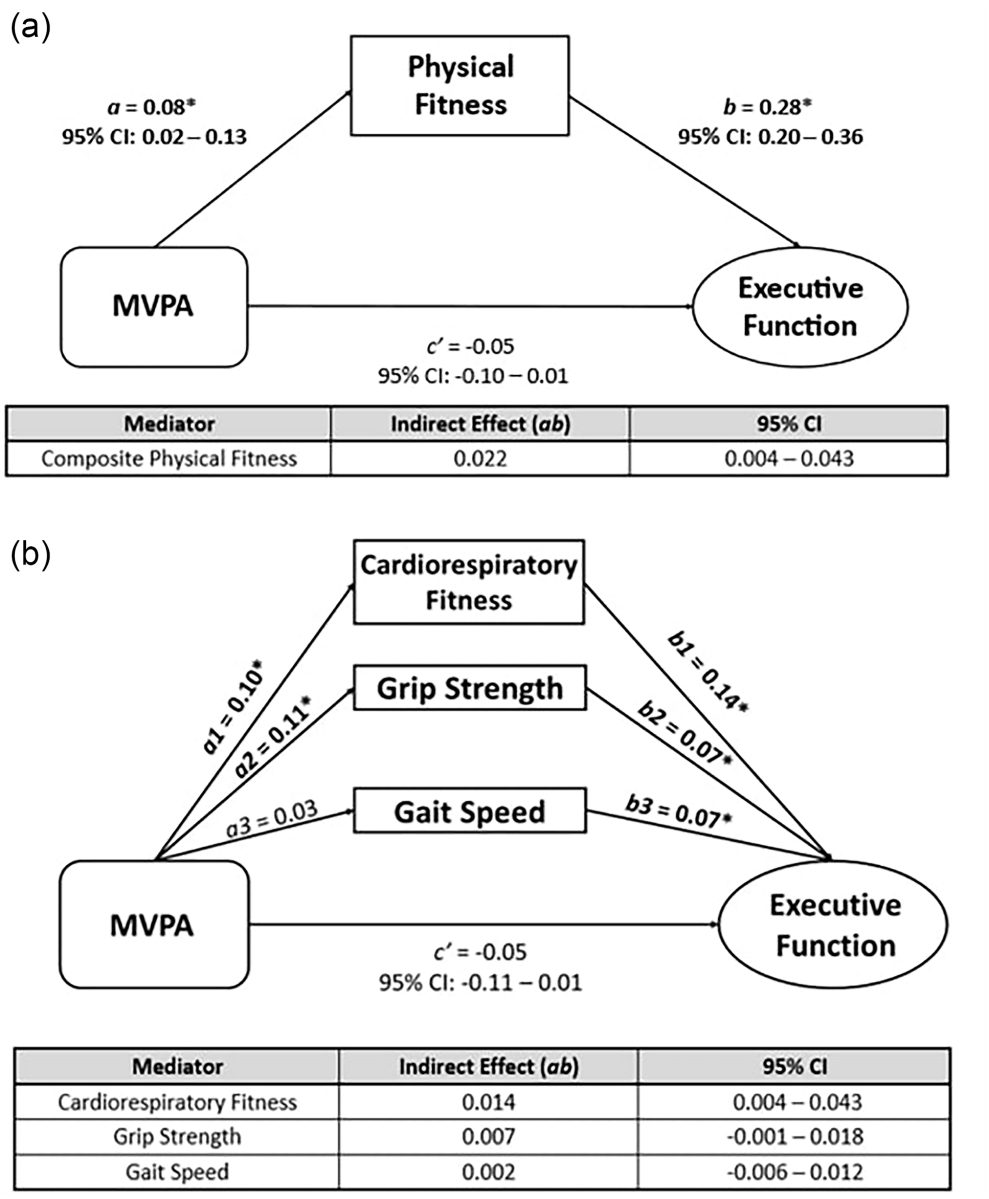
Statistical diagram of the indirect relationship of moderate-to-vigorous physical activity (MVPA) with executive function performance via components of physical fitness. (a) Simple mediation via composite physical fitness. (b) Parallel mediation via components of physical fitness.

The indirect relationship of physical activity with executive function through composite physical fitness is displayed in Figure 3a. Across bootstrap samples, there was a small, positive indirect association of physical activity with executive function performance through higher composite physical fitness (ab = 0.022 (95% CI: 0.004 – 0.043), *p* = .01). The follow-up conditional process analysis indicated that there was not an indirect association of physical activity with executive function through physical fitness in those at the 16^th^ percentile in age (44.4 years; ab = 0.015 (95% CI: -0.011 – 0.046)), whereas there was an indirect relationship for those at the 50^th^ percentile (59.2 years; ab = 0.022 (95% CI: 0.005 – 0.043)) and a qualitatively larger indirect association for those at the 84^th^ percentile (74.1 years; ab = 0.031 (95% CI: 0.003 – 0.066)). However, pairwise contrasts between the conditional indirect effects through the fitness composite score indicated the strength of the indirect effects were not significantly different across age bands (Supplemental Table 2). In a replication analysis with missing data excluded rather than imputed, the small, positive indirect association between physical activity and executive function through composite physical fitness was similar in magnitude but did not reach statistical significance (ab = 0.013 (95% CI: −0.004 – 0.03), *p* = .116). The absence of a significant indirect effect in this subsample was likely due to reduced sample size and changes in sample representativeness in the complete case dataset as described previously.

#### Multiple mediation with components of physical fitness

The indirect association of physical activity with executive functions through the three components of physical fitness is displayed in Figure 3b. Across bootstrap samples, there was a positive indirect relationship of physical activity on executive function performance through cardiorespiratory fitness (ab = 0.014 (95% CI: 0.003 – 0.028)), and there was a trending relationship through grip strength (ab = 0.007 (95% CI: -0.001 – 0.018)), but not gait speed (ab = 0.002 (95% CI: −0.006 – 0.012)). The follow-up conditional process analysis indicated that the indirect relationships through grip strength and gait speed were not dependent on participant age. For cardiorespiratory fitness, there was not an indirect relationship of physical activity on executive functions through cardiorespiratory fitness in those at the 16^th^ percentile in age (44.4 years; ab = 0.009 (95% CI: −0.007 – 0.03)), whereas there was an indirect relationship for those at the 50^th^ (59.2 years; ab = 0.014 (95% CI: 0.003 – 0.028)) and 84^th^ percentile (74.1 years; ab = 0.019 (95% CI: 0.001 – 0.048)). However, pairwise contrasts between the conditional indirect effects through cardiorespiratory fitness indicated the strength of the indirect effects were not significantly different across age bands (Supplemental Table 3).

## Discussion

Few studies have examined the relative associations of physical activity and multiple components of physical fitness with executive function performance across middle age to older adulthood. The results of the present study revealed that physical fitness, but not self-reported moderate-to-vigorous physical activity, was robustly associated with executive function performance in middle-aged and older adults. Furthermore, physical fitness, predominantly related to cardiorespiratory fitness, accounted for a positive indirect association between moderate-to-vigorous physical activity and executive function performance. The indirect association of cardiorespiratory fitness with physical activity and executive function was significant in older study participants (mean age of 59 years or + 1 SD (74 years)), but not younger participants (-1 SD (44 years)), although between-group contrasts were not significant.

Our finding that self-reported moderate-to-vigorous physical activity was not associated with a composite measure of executive function performance was unexpected and not aligned with consensus from extant literature. Several systematic reviews of cross-sectional and longitudinal observational research have provided evidence that greater engagement in physical activity is generally associated with a small, positive effect on cognitive performance across the lifespan and across cognitive domains, including executive function (Cox et al., 2016; Engeroff et al., 2018). However, examples of null or negative associations between self-reported physical activity and executive functions among community-dwelling adults have been reported. For example, a cross-sectional study of cognitively normal older adults found two self-report questionnaires of physical activity to have null and negative associations with composite executive function assessed with the computerized NIH EXAMINER battery (VandeBunte et al., 2022). Additionally, a study of middle-aged adults reported a null association between survey-reported moderate-to-vigorous physical activity and performance on the Symbol Digit Modalities and Digit Span Backwards tasks (Quinlan et al., 2023). Finally, a previous study using the HCP-A sample described inverse trends of similar magnitude found in our study between self-reported total physical activity (including light physical activity) and performance on Trail Making Test A & B (Callow & Smith, 2023). Self-reported physical activity, even on widely used assessments such as the IPAQ-SF, is prone to measurement bias, such as recall and social desirability biases, which may relate to participants’ executive function performance and confound observed relationships (Lee et al., 2011). It is possible that adults with lower cognitive performance overreported their physical activity levels (Herbolsheimer et al., 2018). There may be other subsamples within the study with variable relationships between physical activity and executive function, leading to the overall null physical activity-executive function association observed here.

The finding that participants with higher levels of composite physical fitness tended to perform better on the executive function composite regardless of age was consistent with extant literature, with an effect size comparable to those reported in previous studies examining the relationship between physical fitness and executive function (Callow & Smith, 2023; Weinstein et al., 2012). Additionally, given the well-established negative association between age and executive function performance, it is noteworthy that physical fitness accounted for a similar amount of variance in the model (4%) compared to age (3%). This finding further extends the literature with the inclusion of a sample of middle-aged adults – an age group with little representation in available physical activity-cognition literature – and use of a multidimensional measure of physical fitness. Previous studies have found that higher levels of cardiorespiratory fitness are associated with better performance on diverse measures of executive functions among older adults (S. M. Hayes et al., 2016; Kawagoe et al., 2017; Predovan et al., 2021), but not among young adults (S. M. Hayes et al., 2016). One extant study assessed participants’ physical fitness via cycle ergometry and found their physical work capacity was related to performance on tests of sustained attention, working memory, verbal fluency, and inhibition among those aged 46 – 70 years, but not among those 45 years of age or younger (Gajewski et al., 2023). Additionally, performance on measures of strength (Ahmadi et al., 2024; Frith & Loprinzi, 2018) and gait speed (Kearney et al., 2013; Trapuzzano et al., 2020) have also been associated with executive function performance specifically among older adult samples. There is a more limited number of studies that have included middle-aged adults. One study that included cognitively normal middle-aged to older adults with a similar mean age as our sample found that gait speed was selectively associated with a measure of sustained attention but not other executive functions (Park et al., 2021), suggesting fitness-cognition associations may vary by sample age and cognitive task. To our knowledge, our study is one of the first to demonstrate a robust association between a multidimensional composite of physical fitness and a composite measure of executive function performance across middle age to older adulthood.

Another important aspect of our study was the statistical control for processing speed performance when considering the relationship between modifiable lifestyle variables and executive function. Salthouse (1996), among others, have reported that age-related variance on measures of processing speed account for a substantial amount of the age-related variance in performance on diverse tasks of fluid cognition, including executive function (Albinet et al., 2012). When variance associated with processing speed is accounted for, one has more confidence that observed associations are attributable to the targeted cognitive domain of interest, rather than a more general effect of age-related decline in processing speed. Our finding that processing speed performance was the most robust correlate of executive function performance, explaining an average of 16.5% of the variance, is consistent with the notion of processing speed manifesting effects across multiple cognitive domains in aging. Yet, physical fitness remained significantly associated with executive function performance after controlling for processing speed. This finding emphasizes that physical fitness may have important relationships with components of executive function independent of processing speed, such as the inhibition of prepotent responses, updating of working memory, and ability to flexibly switch perspectives.

The modifiability of physical fitness across the lifespan is determined in part by engagement in physical activity (Liberman et al., 2017). In this study, we demonstrated that engagement in MVPA was associated with higher levels of composite physical fitness and physical fitness in turn related to better executive function performance. This finding was consistent with interventional research suggesting that engagement in aerobic exercise and resistance training programs have beneficial effects on physical fitness that relate to improved cognitive performance, including executive-control processes (Colcombe & Kramer, 2003; Erickson et al., 2011; Liu-Ambrose et al., 2010). Additionally, combining aerobic exercise interventions with cognitive training can improve attention and processing speed, but does not seem to improve cardiorespiratory fitness or cognition above and beyond aerobic exercise alone (Roig-Coll et al., 2020). However, less is known about the potential variables connecting unstructured forms of physical activity with cognitive function. Here, we demonstrated that self-reported lifestyle-embedded MVPA related to executive function primarily via cardiorespiratory fitness, and marginally via muscular strength, but not through speed. One previous investigation among healthy adults aged 50 – 70 found that cardiorespiratory fitness, assessed via a 1-mile walk test, mediated an association between self-reported sport-related physical activity and executive function and attention-speed composites (Castells Sánchez et al., 2021). This indirect association through cardiorespiratory fitness was only significant in men and non-sportive forms of physical activity were not associated with cognition (Castells Sánchez et al., 2021). Another observational study suggested that hand grip strength and gait speed partially mediated the relationship between age and cognitive status assessed with the Mini-Mental State Examination among Columbian older adults (Pérez-Sousa et al., 2021). Our study adds to this literature by illustrating the importance of cardiorespiratory fitness, above and beyond other fitness measures, along with MVPA for preserving executive function performance from middle age to older adulthood.

This study had limitations. The data were cross-sectional, which precludes any conclusion of causal relationships. Our data suggest that physical activity may relate to executive function through its joint association with physical fitness, but a longitudinal study design with measures repeated at different time points would provide stronger evidence for mediation following temporal ordering. Second, HCP used a self-reported measure of physical activity, which may be influenced by age, education, and cultural factors, and could be prone to recall and social desirability biases that interact with cognitive ability. However, to partially address this limitation, we analyzed moderate-to-vigorous forms of physical activity as they show stronger relationships with physical fitness and may be easier to recall than lighter incidental forms of activity, such as walking. Third, the total MVPA variable is an absolute measure of physical activity engagement and does not consider individual factors such as body weight, sex, and fitness level, which may influence the association between physical activity and cognition. We did not explore potentially relevant modifiers, such as participant sex, in the relationships between physical activity, physical fitness, and executive function. Fourth, we used a composite measure of executive function for statistical efficiency and to maximize generalizability to extant literature and therefore were not able to examine whether subdomains of executive function have differential associations with physical activity and physical fitness. Finally, the inclusion of gait speed, which is often considered a measure of physical function, as a measure of physical fitness was debatable. The authors acknowledge that assessment of preferred gait speed, rather than maximal gait speed, may not represent one’s maximal capacity. Nevertheless, when a data-driven approach (confirmatory factor analysis) was implemented, preferred gait speed loaded significantly on the latent fitness variable.

This study also had a few notable strengths. First, the HCP-A included a large, demographically diverse sample of middle-aged to older American adults. Middle-aged adults are an underrepresented age group in the physical activity-cognition literature, and the results of this study suggest that the benefits of physical activity and physical fitness on cognition may begin in later middle adulthood. Second, the HCP utilized widely used and validated assessments of executive function performance from the NIH Toolbox. We opted to average performance across the three canonical subcomponents of executive function to increase the reliability of our results and generalizability to extant literature. Additionally, statistical control for processing speed performance enhanced the rigor of the approach by ruling out a potential confound in the relationship between modifiable lifestyle variables and executive function. Moreover, when assessing the impact of physical fitness, we controlled for physical activity, which further improved the methodological rigor. Finally, we explored the multidimensional construct of physical fitness. The extant literature is weighted toward the assessment of individual components of fitness but emerging evidence suggests multiple aspects of fitness are related and may be important indicators of cognitive performance. Although cardiorespiratory fitness was most strongly associated with executive function, strength was also significantly associated with executive function, albeit to a lesser degree. Moreover, the composite measure of fitness accounted for more variance in executive function than any single fitness metric, highlighting the importance of evaluating multiple components of fitness. However, given the unequal contributions of the physical fitness components to executive function, further refinement of the construct of physical fitness and examination of the relative importance of fitness measures to cognition is warranted.

In conclusion, the current findings demonstrate that measures of physical fitness, primarily cardiorespiratory fitness, have significant direct relationships with executive function performance, and partly explain a relationship between self-reported moderate-to-vigorous physical activity and executive function performance. These findings suggest that maintained or improved physical fitness through engagement in physical activity may be an increasingly important contributor to cognitive maintenance.

## Supporting information

Stauder et al. supplementary material Stauder et al. supplementary material

## Data Availability

The HCP-Aging 2.0 Release data used in this report came from https://doi.org/10.15154/1520707. Code used to generate the final dataset and perform analyses for this study are available upon reasonable request. The study reported within this manuscript was not preregistered.

## Supplementary material

The supplementary material for this article can be found at https://doi.org/10.1017/S1355617725101446.

## References

[ref1] Ahmadi, S. , Quirion, I. , Faivre, P. , Registe, P. P. W. , O’Brien, M. W. , Bray, N. W. , Dupuy, O. , Sénéchal, M. , Bélanger, M. , & Mekari, S. (2024). Association between physical fitness and executive functions in cognitively healthy female older adults: A cross-sectional study.GeroScience. 46, 5701–5710. 10.1007/s11357-024-01188-y 38722469 PMC11494617

[ref2] Albinet, C. T. , Boucard, G. , Bouquet, C. A. , & Audiffren, M. (2012). Processing speed and executive functions in cognitive aging: How to disentangle their mutual relationship? Brain and Cognition, 79(1), 1–11. 10.1016/j.bandc.2012.02.001 22387275

[ref3] Blondell, S. J. , Hammersley-Mather, R. , & Veerman, J. L. (2014). Does physical activity prevent cognitive decline and dementia?: A systematic review and meta-analysis of longitudinal studies. BMC Public Health, 14(1), 510. 10.1186/1471-2458-14-510 24885250 PMC4064273

[ref4] Bookheimer, S. Y. , Salat, D. H. , Terpstra, M. , Ances, B. M. , Barch, D. M. , Buckner, R. L. , Burgess, G. C. , Curtiss, S. W. , Diaz-Santos, M. , Elam, J. S. , Fischl, B. , Greve, D. N. , Hagy, H. A. , Harms, M. P. , Hatch, O. M. , Hedden, T. , Hodge, C. , Japardi, K. C. , Kuhn, T. P. , & E., Yacoub (2019). The lifespan human connectome project in aging: An overview. NeuroImage, 185, 335–348. 10.1016/j.neuroimage.2018.10.009 30332613 PMC6649668

[ref5] Brothers, S. L. , & Suchy, Y. (2022). Daily assessment of executive functioning and expressive suppression predict daily functioning among community-dwelling older adults. Journal of the International Neuropsychological Society, 28(9), 974–983. 10.1017/S1355617721001156 34666858

[ref6] Buuren, Svan , Groothuis-Oudshoorn, K. (2011). Mice: Multivariate imputation by chained equations in R. Journal of Statistical Software, 45, 1–67. 10.18637/jss.v045.i03

[ref7] Callow, D. D. , & Smith, J. C. (2023). Physical fitness, cognition, and structural network efficiency of brain connections across the lifespan. Neuropsychologia, 182, 108527. 10.1016/j.neuropsychologia.2023.108527 36871816 PMC12681381

[ref8] Carson, N. , Leach, L. , & Murphy, K. J. (2018). A re-examination of montreal cognitive assessment (MoCA) cutoff scores. International Journal of Geriatric Psychiatry, 33(2), 379–388. 10.1002/gps.4756 28731508

[ref9] Caspersen, C. J. , Powell, K. E. , & Christenson, G. M. (1985). Physical activity, exercise, and physical fitness: Definitions and distinctions for health-related research. Public Health Reports, 100(2), 126–131. 3920711 PMC1424733

[ref10] Castells Sánchez, A. , Roig Coll, F. , Lamonja Vicente, N. , Torán Monserrat, P. , Pera, G. , Montero, P. , Dacosta Aguayo, R. , Bermudo Gallaguet, A. , Bherer, L. , Erickson, K. , Mataró Serrat, M. (2021). Sex matters in the association between physical activity and fitness with cognition. Articles Publicats En Revistes (Psicologia Clínica i Psicobiologia), 53(6), 1252–1259. https://diposit.ub.edu/dspace/handle/2445/218645 10.1249/MSS.0000000000002570 33394900

[ref11] Colcombe, S. , & Kramer, A. F. (2003). Fitness effects on the cognitive function of older adults: A meta-analytic study. Psychological Science, 14(2), 125–130. 10.1111/1467-9280.t01-1-01430 12661673

[ref12] Cox, E. P. , O’Dwyer, N. , Cook, R. , Vetter, M. , Cheng, H. L. , Rooney, K. , & O’Connor, H. (2016). Relationship between physical activity and cognitive function in apparently healthy young to middle-aged adults: A systematic review. Journal of Science and Medicine in Sport, 19(8), 616–628. 10.1016/j.jsams.2015.09.003 26552574

[ref13] Craig, C. L. , Marshall, A. L. , Sjöström, M. , Bauman, A. E. , Booth, M. L. , Ainsworth, B. E. , Pratt,M. I. C. H. A. E. L. , Ekelund,U. L. F. , A. G. N. E. T. A.,Yngve , Sallis,J. A. M. E. S. F. , & Oja, P. (2003). International physical activity questionnaire: 12-country reliability and validity. Medicine & Science in Sports & Exercise, 35(8), 1381–1395. 10.1249/01.MSS.0000078924.61453.FB 12900694

[ref14] Diamond, A. (2013). Executive functions. Annual Review of Psychology, 64(1), 135–168. 10.1146/annurev-psych-113011-143750 PMC4084861 23020641

[ref15] Dumurgier, J. , Artaud, F. , Touraine, C. , Rouaud, O. , Tavernier, B. , Dufouil, C. , Singh-Manoux, A. , Tzourio, C. , Elbaz, A. (2017). Gait speed and decline in gait speed as predictors of incident Dementia. The Journals of Gerontology: Series A, 72(5), 655–661. 10.1093/gerona/glw110 27302701

[ref16] Engeroff, T. , Ingmann, T. , & Banzer, W. (2018). Physical activity throughout the adult life span and domain-specific cognitive function in old age: A systematic review of cross-sectional and longitudinal data. Sports Medicine, 48(6), 1405–1436. 10.1007/s40279-018-0920-6 29667159

[ref17] Eppinger-Ruiz de Zarate, A. , Powell, D. , Kühnhausen, J. , Allan, J. L. , Johnstone, A. , Crabtree, D. R. , Buosi, W. , Fyfe, C. L. , McMinn, D. , McCavour, B. , Gawrilow, C. , & Stadler, G. (2024). Free-living physical activity and executive function: A multi-study analysis of age groups and times of day. International Journal of Clinical and Health Psychology, 24(1), 100425. 10.1016/j.ijchp.2023.100425 38089542 PMC10714236

[ref18] Erickson, K. I. , Hillman, C. , Stillman, C. M. , Ballard, R. M. , Bloodgood, B. , Conroy, D. E. , Macko, R. , Marquez, D. , Petruzzello, S. , & Powell, K. E. (2019). Physical activity, cognition, and brain outcomes: A review of the 2018 physical activity guidelines. Medicine and Science in Sports and Exercise, 51(6), 1242–1251. 10.1249/MSS.0000000000001936 31095081 PMC6527141

[ref19] Erickson, K. I. , Voss, M. W. , Prakash, R. S. , Basak, C. , Szabo, A. , Chaddock, L. , Kim, J. S. , Heo, S. , Alves, H. , White, S. M. , Wojcicki, T. R. , Mailey, E. , Vieira, V. J. , Martin, S. A. , Pence, B. D. , Woods, J. A. , McAuley, E. , & Kramer, A. F. (2011). Exercise training increases size of hippocampus and improves memory. Proceedings of The National Academy of Sciences of The United States of America, 108(7), 3017–3022. 10.1073/pnas.1015950108 21282661 PMC3041121

[ref20] Ferreira, M. L. , Sherrington, C. , Smith, K. , Carswell, P. , Bell, R. , Bell, M. , Nascimento, D. P. , Máximo Pereira, L. S. , & Vardon, P. (2012). Physical activity improves strength, balance and endurance in adults aged 40-65 years: A systematic review. Journal of Physiotherapy, 58(3), 145–156. 10.1016/S1836-9553(12)70105-4 22884181

[ref21] Frith, E. , & Loprinzi, P. D. (2018). The association between lower extremity muscular strength and cognitive function in a national sample of older adults. Journal of Lifestyle Medicine, 8(2), 99–104. 10.15280/jlm.2018.8.2.99 30474005 PMC6239135

[ref22] Gajewski, P. D. , Golka, K. , Hengstler, J. G. , Kadhum, T. , Digutsch, J. , Genç, E. , Wascher, E. , & Getzmann, S. (2023). Does physical fitness affect cognitive functions differently across adulthood? An advantage of being older. Frontiers in Psychology, 14, 1134770. 10.3389/fpsyg.2023.1134770 37397318 PMC10312084

[ref23] Groemping, U. (2007). Relative importance for linear regression in R: The package relaimpo. Journal of Statistical Software, 17, 1–27.

[ref24] Hayes, A. F. (2023). Introduction to mediation, moderation, and conditional process analysis: Third Edition: A Regression-Based Approach. Guilford Press. https://www.guilford.com/books/Introduction-to-Mediation-Moderation-and-Conditional-Process-Analysis/Andrew-Hayes/9781462549030

[ref25] Hayes, S. M. , Forman, D. E. , & Verfaellie, M. (2016). Cardiorespiratory fitness is associated with cognitive performance in older but not younger adults. The Journals of Gerontology: Series B, 71(3), 474–482. 10.1093/geronb/gbu167 PMC6959383 25528256

[ref26] Herbolsheimer, F. , Riepe, M. W. , & Peter, R. (2018). Cognitive function and the agreement between self-reported and accelerometer-accessed physical activity. BMC Geriatrics, 18(1), 56. 10.1186/s12877-018-0747-x 29466954 PMC5822490

[ref27] Igartua, J.-J. , & Hayes, A. F. (2021). Mediation, moderation, and conditional process analysis: Concepts, computations, and some common confusions. The Spanish Journal of Psychology, 24, e49. 10.1017/SJP.2021.46 35923144

[ref28] Kawagoe, T. , Onoda, K. , & Yamaguchi, S. (2017). Associations among executive function, cardiorespiratory fitness, and brain network properties in older adults. Scientific Reports, 7(1), Article 1. 10.1038/srep40107 28054664 PMC5215211

[ref29] Kearney, F. C. , Harwood, R. H. , Gladman, J. R. F. , Lincoln, N. , & Masud, T. (2013). The relationship between executive function and falls and gait Abnormalities in older adults: A systematic review. Dementia and Geriatric Cognitive Disorders, 36(1-2), 20–35. 10.1159/000350031 23712088

[ref30] Laera, G. , Joly-Burra, E. , Zuber, S. , Ballhausen, N. , Künzi, M. , Ihle, A. , da Silva Coelho, C. , Haas, M. , Mikneviciute, G. , Tinello, D. , Kliegel, M. , & Hering, A. (2023). Do executive functions explain older adults’ health-related quality of life beyond event-based prospective memory? Aging, Neuropsychology, and Cognition, 30(2), 135–149. 10.1080/13825585.2021.1989368 34665685

[ref31] Lee, P. H. , Macfarlane, D. J. , Lam, T. , & Stewart, S. M. (2011). Validity of the international physical activity questionnaire short form (IPAQ-SF): A systematic review. International Journal of Behavioral Nutrition and Physical Activity, 8(1), 115. 10.1186/1479-5868-8-115 22018588 PMC3214824

[ref32] Liberman, K. , Forti, L. N. , Beyer, I. , & Bautmans, I. (2017). The effects of exercise on muscle strength, body composition, physical functioning and the inflammatory profile of older adults: A systematic review. Current Opinion in Clinical Nutrition & Metabolic Care, 20(1), 30. 10.1097/MCO.0000000000000335 27755209

[ref33] Liu-Ambrose, T. , Nagamatsu, L. S. , Graf, P. , Beattie, B. L. , Ashe, M. C. , & Handy, T. C. (2010). Resistance training and executive functions: A 12-month randomized controlled trial. Archives of Internal Medicine, 170(2), 170–178. 10.1001/archinternmed.2009.494 20101012 PMC3448565

[ref34] Luszcz, M. (2011). Chapter 4—Executive Function and Cognitive Aging. In K. W. Schaie , & S. L. Willis (Eds.), Handbook of the Psychology of Aging (Seventh Edition) (pp. 59–72). Academic Press, 10.1016/B978-0-12-380882-0.00004-8

[ref35] Mielke, M. M. , Roberts, R. O. , Savica, R. , Cha, R. , Drubach, D. I. , Christianson, T. , Pankratz, V. S. , Geda, Y. E. , Machulda, M. M. , Ivnik, R. J. , Knopman, D. S. , Boeve, B. F. , Rocca, W. A. , & Petersen, R. C. (2013). Assessing the temporal relationship between cognition and gait: Slow gait predicts cognitive decline in the mayo clinic study of aging. The Journals of Gerontology: Series A, 68(8), 929–937. 10.1093/gerona/gls256 PMC3712358 23250002

[ref36] Miyake, A. , Friedman, N. P. , Emerson, M. J. , Witzki, A. H. , Howerter, A. , & Wager, T. D. (2000). The unity and diversity of executive functions and their contributions to complex “Frontal Lobe” tasks: A latent variable analysis. Cognitive Psychology, 41(1), 49–100. 10.1006/cogp.1999.0734 10945922

[ref37] Nasreddine, Z. S. , Phillips, N. A. , Bédirian, Vérie , Charbonneau, S. , Whitehead, V. , Collin, I. , Cummings, J. L. , & Chertkow, H. (2005). The montreal cognitive assessment, moCA: A brief screening tool for mild cognitive impairment. Journal of the American Geriatrics Society, 53(4), 695–699. 10.1111/j.1532-5415.2005.53221.x 15817019

[ref38] Nilsson, J. , Ekblom, M. , & Lövdén, M. (2022). Associations of cardiorespiratory fitness and moderate-to-vigorous physical activity with latent cognitive abilities in older adults. Psychology of Sport and Exercise, 60, 102171. 10.1016/j.psychsport.2022.102171

[ref39] O’Brien, M. W. , Kimmerly, D. S. , & Mekari, S. (2021). Greater habitual moderate-to-vigorous physical activity is associated with better executive function and higher prefrontal oxygenation in older adults. GeroScience, 43(6), 2707–2718. 10.1007/s11357-021-00391-5 34081258 PMC8602604

[ref41] Park, H. , Aul, C. , DeGutis, J. , Lo, O.-Y. , Poole, V. N. , McGlinchey, R. , Bean, J. F. , Leritz, E. , & Esterman, M. (2021). Evidence for a specific association between sustained attention and gait speed in middle-to-older-aged adults. Frontiers in Aging Neuroscience, 13, 703434. 10.3389/fnagi.2021.703434 34290601 PMC8289388

[ref42] Pérez-Sousa, M.Á. , del Pozo-Cruz, J. , Olivares, P. R. , Cano-Gutiérrez, C. A. , Izquierdo, M. , & Ramírez-Vélez, R. (2021). Role for physical fitness in the association between age and cognitive function in older adults: A mediation analysis of the SABE Colombia study. International Journal of Environmental Research and Public Health, 18(2), 751. 10.3390/ijerph18020751 33477293 PMC7829928

[ref43] Prakash, R. S. , Voss, M. W. , Erickson, K. I. , & Kramer, A. F. (2015). Physical activity and cognitive vitality. Annual Review of Psychology, 66, 769–797. 10.1146/annurev-psych-010814-015249 25251492

[ref44] Predovan, D. , Berryman, N. , Lussier, M. , Comte, F. , Vu, T. T. M. , Villalpando, J. M. , & Bherer, L. (2021). Assessment of the relationship between executive function and cardiorespiratory fitness in healthy older adults. Frontiers in Psychology, 12, 742184. 10.3389/fpsyg.2021.742184 34803824 PMC8595132

[ref45] Quinlan, C. , Rattray, B. , Pryor, D. , Northey, J. M. , & Cherbuin, N. (2023). Physical activity and cognitive function in middle-aged adults: A cross-sectional analysis of the PATH through life study. Frontiers in Psychology, 14, 1022868. 10.3389/fpsyg.2023.1022868 37691791 PMC10484531

[ref46] R Core Team (2021). R: A language and environment for statistical computing https://www.R-project.org/.

[ref47] Reuben, D. B. , Magasi, S. , McCreath, H. E. , Bohannon, R. W. , Wang, Y.-C. , Bubela, D. J. , Rymer, W. Z. , Beaumont, J. , Rine, R. M. , Lai, J.-S. , Gershon, R. C. (2013). Motor assessment using the NIH toolbox. Neurology, 80(11_supplement_3), S65–S75. 10.1212/WNL.0b013e3182872e01 23479547 PMC3662336

[ref48] Reuter-Lorenz, P. A. , Festini, S. B. , & Jantz, T. K. (2021). Chapter 5—Executive functions and neurocognitive aging. In K. W. Schaie , & S. L. Willis (Eds.), Handbook of the Psychology of Aging (Ninth Edition) (pp. 67–81). Academic Press, 10.1016/B978-0-12-816094-7.00019-2

[ref49] Roig-Coll, F. , Castells-Sánchez, A. , Lamonja-Vicente, N. , Torán-Monserrat, P. , Pera, G. , García-Molina, A. , Tormos, J. M. , Montero-Alía, P. , Alzamora, M. T. , Dacosta-Aguayo, R. D. , Soriano-Raya, J. J. , Cáceres, C. , Erickson, K. I. , Mataró, M. (2020). Effects of aerobic exercise, cognitive and combined training on cognition in physically inactive healthy late-middle-aged adults: The projecte moviment randomized controlled trial. Frontiers in Aging Neuroscience, 12, 590168. 10.3389/fnagi.2020.590168 33192485 PMC7664521

[ref50] Salthouse, T. A. (1996). The processing-speed theory of adult age differences in cognition. Psychological Review, 103(3), 403–428. 10.1037/0033-295X.103.3.403 8759042

[ref51] Sjostrom, M. , Ainsworth, B. , Bauman, A. , Bull, F. , Hamilton-Craig, C. , & Sallis, J. (2005). Guidelines for data processing analysis of the international physical activity questionnaire (IPAQ)—Short and long forms. https://api.semanticscholar.org/CorpusID:79242415

[ref52] Smith, S. C. (2007). Multiple risk factors for cardiovascular disease and diabetes mellitus. The American Journal of Medicine, 120(3, Supplement 1), S3–S11. 10.1016/j.amjmed.2007.01.002 17320520

[ref53] Sprague, B. N. , Phillips, C. B. , & Ross, L. A. (2019). Age-varying relationships between physical function and cognition in older adulthood. The Journals of Gerontology Series B: Psychological Sciences and Social Sciences, 74(5), 772–784. 10.1093/geronb/gbx126 29121330 PMC6566327

[ref54] Stauder, M. , Hiersche, K. J. , & Hayes, S. M. (2024). Examining cross-sectional and longitudinal relationships between multidomain physical fitness metrics, education, and cognition in black older adults. Aging, Neuropsychology, and Cognition, 31(4), 646–660. 10.1080/13825585.2023.2225848 PMC10739568 37345613

[ref55] Trapuzzano, A. , Chizmar, S. , Wilda, L. , & Dawson, N. (2020). What makes us walk: Predictors and the interplay of physical and cognitive factors on gait speed in community dwelling older adults. OBM Geriatrics, 4(3), 1–15. 10.21926/obm.geriatr.2003134

[ref56] Van Abbema, R. , De Greef, M. , Crajé, C. , Krijnen, W. , Hobbelen, H. , & Van Der Schans, C. (2015). What type, or combination of exercise can improve preferred gait speed in older adults? A meta-analysis. BMC Geriatrics, 15(1), 72. 10.1186/s12877-015-0061-9 26126532 PMC4488060

[ref57] VandeBunte, A. , Gontrum, E. , Goldberger, L. , Fonseca, C. , Djukic, N. , You, M. , Kramer, J. H. , & Casaletto, K. B. (2022). Physical activity measurement in older adults: Wearables versus self-report. Frontiers in Digital Health, 4, 869790. 10.3389/fdgth.2022.869790 36120711 PMC9470756

[ref58] Vanhees, L. , Lefevre, J. , Philippaerts, R. , Martens, M. , Huygens, W. , Troosters, T. , & Beunen, G. (2005). How to assess physical activity? How to assess physical fitness? European Journal of Cardiovascular Prevention and Rehabilitation: Official Journal of the European Society of Cardiology, Working Groups on Epidemiology & Prevention and Cardiac Rehabilitation and Exercise Physiology, 12(2), 102–114. 10.1097/01.hjr.0000161551.73095.9c 15785295

[ref59] Wang, R. , Ekblom, M. M. , Arvidsson, D. , Fridolfsson, J. , Börjesson, M. , & Ekblom, Ö. (2022). The interrelationship between physical activity intensity, cardiorespiratory fitness, and executive function in middle-aged adults: An observational study of office workers. Frontiers in Public Health, 10, 1035521. 10.3389/fpubh.2022.1035521 36438224 PMC9682261

[ref60] Weinstein, A. M. , Voss, M. W. , Prakash, R. S. , Chaddock, L. , Szabo, A. , White, S. M. , Wojcicki, T. R. , Mailey, E. , McAuley, E. , Kramer, A. F. , Erickson, K. I. (2012). The association between aerobic fitness and executive function is mediated by prefrontal cortex volume. Brain, Behavior, and Immunity, 26(5), 811–819. 10.1016/j.bbi.2011.11.008 22172477 PMC3321393

[ref61] Weintraub, S. , Dikmen, S. S. , Heaton, R. K. , Tulsky, D. S. , Zelazo, P. D. , Bauer, P. J. , Carlozzi, N. E. , Slotkin, J. , Blitz, D. , Wallner-Allen, K. , Fox, N. A. , Beaumont, J. L. , Mungas, D. , Nowinski, C. J. , Richler, J. , Deocampo, J. A. , Anderson, J. E. , Manly, J. J. , Borosh, B. … Gershon, R. C. (2013). Cognition assessment using the NIH toolbox. Neurology, 80(11 Supplement 3), S54–S64. 10.1212/WNL.0b013e3182872ded 23479546 PMC3662346

[ref62] Won, J. , Callow, D. D. , Purcell, J. J. , & Smith, J. C. (2024). Hippocampal functional connectivity mediates the association between cardiorespiratory fitness and cognitive function in healthy young adults. Journal of the International Neuropsychological Society, 30(3), 199–208. 10.1017/S1355617723000498 37646336

[ref63] Wu, Z. , Woods, R. L. , Chong, T. T.‐J. , Orchard, S. G. , Shah, R. C. , Wolfe, R. , Storey, E. , Sheets, K. M. , Murray, A. M. , McNeil, J. J. , & Ryan, J. (2023). Grip strength, gait speed, and trajectories of cognitive function in community-dwelling older adults: A prospective study. Alzheimer’s & Dementia: Diagnosis, Assessment & Disease Monitoring, 15(1), e12388. 10.1002/dad2.12388 PMC9927855 36815873

