# Supplementary material for: The role of age and physical fitness on the relationship between physical activity and executive function
Source: J Int Neuropsychol Soc. 2025 Oct 23;31(7-8):479–89. doi: 10.1017/S1355617725101446 (PMC12683824; doi:10.1017/S1355617725101446)
Supplement: Stauder et al. supplementary material [file S1355617725101446sup001.docx]

| **Supplemental Table 1.** Proportion of data imputed by variable. | |
| --- | --- |
| Variable | Percent of Data Imputed (%) |
| Serum Glucose | 12.18 |
| 2-minute Walk Test | 3.85 |
| 4-meter Walk Test | 2.08 |
| Systolic Blood Pressure | 0.96 |
| Diastolic Blood Pressure | 0.96 |
| Grip Strength | 0.32 |
| Years of Education | 0.16 |
| *Note.* Any variable not listed had complete data. | |

| **Supplemental Table 2.** Pairwise contrasts between conditional indirect effects of physical activity on executive function through composite fitness by age band. | | | | | |  |
| --- | --- | --- | --- | --- | --- | --- |
| Age Band Effect Comparison | Effect Size  1 | Effect Size  2 | Effect Size Contrast (1-2) | Standard Error | 95% Confidence Interval | |
| MA-YA | 0.022 | 0.015 | 0.007 | 0.010 | -0.014 – 0.027 | |
| OA-YA | 0.031 | 0.015 | 0.016 | 0.023 | -0.029 – 0.062 | |
| OA-MA | 0.031 | 0.022 | 0.009 | 0.013 | -0.016 – 0.036 | |
| *Note.* YA = younger adult age band (mean age = 44.4 years), MA = middle-aged adult (mean age = 59.2 years), OA = older adult (mean age = 74.1) | | | | | |  |

| **Supplemental Table 3.** Pairwise contrasts between conditional indirect effects of physical activity on executive function through cardiorespiratory fitness by age band. | | | | | |  |
| --- | --- | --- | --- | --- | --- | --- |
| Age Band Effect Comparison | Effect Size  1 | Effect Size  2 | Effect Size Contrast (1-2) | Standard Error | 95% Confidence Interval | |
| MA-YA | 0.014 | 0.009 | 0.005 | 0.008 | -0.010 – 0.021 | |
| OA-YA | 0.019 | 0.009 | 0.009 | 0.016 | -0.020 – 0.045 | |
| OA-MA | 0.019 | 0.014 | 0.005 | 0.009 | -0.010 – 0.026 | |
| *Note.* YA = younger adult age band (mean age = 44.4 years), MA = middle-aged adult (mean age = 59.2 years), OA = older adult (mean age = 74.1) | | | | | |  |
